# Treatment of obesity by acupuncture combined with medicine based on pathophysiological mechanism: A review

**DOI:** 10.1097/MD.0000000000036071

**Published:** 2023-12-01

**Authors:** Shiyu Niu, Lihong Ren

**Affiliations:** a Second Affiliated Hospital of Heilongjiang Traditional Chinese Medicine, Harbin, Heilongjiang Province; b The Second Hospital of Harbin, Harbin, Heilongjiang Province.

**Keywords:** combination of acupuncture and medicine, epigenetics, obesity, pathophysiology

## Abstract

Obesity is a complex, multifactorial disease. The incidence of overweight and obesity has doubled worldwide since 1980, and nearly one-third of the world population is now classified as overweight or obese. Obesity rates are increasing in all age groups and for both sexes, regardless of geographic region, race, or socioeconomic status, although they are generally higher in older adults and women. Although the absolute prevalence of overweight and obesity varies widely, this trend is similar across different regions and countries. In some developed countries, the prevalence of obesity has levelled off over the past few years. However, obesity has become a health problem that cannot be ignored in low- and middle-income countries. Although the drug treatment model of modern medicine has a significant therapeutic effect in the treatment of obesity, its adverse effects are also obvious. Acupuncture combined with Chinese medicine treatment of obesity has prominent advantages in terms of clinical efficacy, and its clinical safety is higher, with fewer adverse reactions. The combination of acupuncture and medicine in the treatment of obesity is worth exploring.

## 1. Introduction

Since 1980, there has been a significant increase in obesity worldwide, with the incidence of overweight and obesity doubling; thus, nearly one-third of the world population is now classified as overweight or obese.^[1]^ Obesity refers to a person body mass index (BMI); overweight refers to a BMI of 25.0 to 29.9, and obesity refers to a BMI ≥ 30. Obesity increases the likelihood of various diseases and conditions, including type 2 diabetes mellitus, hyperlipidemia, hypertension, cardiovascular disease, chronic kidney disease, metabolic syndrome, obstructive sleep apnea, osteoporosis, and depression.^[2]^ All of these factors have a negative impact on patient quality of life, productivity, and healthcare costs. The medical costs of obese patients are estimated to be 30% higher than those of obese patients.^[3]^ The consequences of treating obesity are expensive price to pay for patients, as the associated total medical costs double every decade.^[4]^

According to the latest research, the quality of food sources and nutrients is more important than their quantity for weight control and disease prevention.^[5]^ In addition, genetic factors play a key role in determining an individual propensity to gain weight.^[6]^ Recent epigenetic studies have provided a useful tool for understanding the increase in obesity worldwide.^[7]^ With the continuous progress of many new scientific studies, obesity is rapidly being recognized scientifically by the academic community. In this article, we will discuss the epidemiology, pathophysiology, and genetics of obesity and the combination of Chinese medicine and acupuncture.

### 1.1. Epidemiology of obesity

Globally, an estimated 1.9 billion adults were overweight in 2015 and 6.09 one billion adults were obese, totaling approximately 39% of the world population.^[8]^ Among young adults (20–44 years), the prevalence of overweight in women is somewhat lower than that in men; however, this trend is reversed after 45 to 49 years, perhaps coinciding with menopause in women. In other age groups, the prevalence of obesity in women is generally higher than that in men, with the largest sex difference between 50 and 65 years.^[9]^ In China, a study based on 22 years of surveillance of 12,543 participants showed that the age-adjusted prevalence of obesity increased from 2.15% to 13.99% for both sexes, from 2.78% to 13.22% for females, and from 1.46% to 14.99% for males.^[10,11]^

The Americas and Europe have the highest prevalence of overweight and obesity. In the Americas, the prevalence of overweight increased from 45.3% in 1980 to 64.2% in 2015, while the prevalence of obesity increased from 12.9% in 1980 to 28.3% in 2015.^[12]^ The United States and Mexico have the highest prevalence of overweight and obesity, respectively. In Europe, the prevalence of overweight increased from 48% in 1980 to 59.6% in 2015, and the prevalence of obesity increased from 14.5% in 1980 to 22.9% in 2015.^[12]^

The prevalence of overweight and obesity is increasing in low- and middle-income countries, particularly urban areas. Since 2000, the overweight rate among African children under the age of 5 years has increased by 24%. As of 2019, almost half of Asian children under the age of 5 are obese or overweight.^[13]^

### 1.2. Pathophysiological mechanisms of obesity

The root cause of obesity is a long-term energy imbalance; that is, too many calories are consumed and too few calories are consumed. During the course of evolution, humans and their ancestors had to live in a period of undernourishment. Thus, it is likely that selection pressure contributed to the genotype favoring overeating, low energy expenditure, and inactivity.^[14]^

Biomedical researchers are exploring the biological mechanisms that lead to obesity; however, the underlying causes remain controversial. The obesity epidemic is largely driven by energy gains from high-return, high-energy foods. Diet and various social, economic, and environmental factors related to food supply have a strong impact on patients’ ability to achieve balance.^[15]^ In a 13-year follow-up study of 3000 young people, it was found that those who consumed more fast food had an average of 6 kg more and a larger waistline than those with the lowest fast food intake. They were also found to have a higher incidence of weight-related negative health problems, such as elevated triglycerides, and twice the incidence of metabolic syndrome.^[16]^ These issues are compounded in certain individuals who possess a genetic predisposition to fat accumulation, which may be caused by an important interaction between the balance circuitry and brain reward. Accumulation of lipid metabolites, inflammatory signals, or other mechanisms of hypothalamic neuronal damage may also contribute to obesity, which may explain the biological defense against elevated body fat mass.^[17]^

These studies have improved our understanding of how food cravings are disturbed in the brains of obese patients; how adipose tissue, gut, or estrogen hormones regulate appetite and satiety in the hypothalamus; and how dysfunction of adipose tissue can be secondary to health problems.^[18,19]^ The critical role of certain brain regions in regulating body weight has become apparent from the observation of abnormal foraging behavior and obesity in animals with lesions and in humans with tumors affecting the hypothalamus.^[20,21]^

Human knowledge of the gut microbiome has greatly improved in recent years, as has the understanding of its complex relationship with diseases. The human body contains approximately 3.8 × 1013 species of microorganisms, most of which occupy the gastrointestinal tract. More than half of the microorganisms are bacteria, followed by archaea and eukaryotes.^[22]^ Normally, the gut microbiota has substantial beneficial effects on the host, including involvement in carbohydrate and lipid metabolism, synthesis of vitamins and amino acids, epithelial cell proliferation, protection against pathogens, and regulation of hormones.^[23]^ An imbalance in microbial populations has been shown to be associated with a wide range of diseases, including neurological disorders, inflammatory bowel disease, malnutrition, diabetes, and obesity.^[24]^ Recent studies have shown that calorie restriction helps to reshape the intestinal microbiota, whereas the use of antibiotics can adversely affect the intestinal microbiota, leading to obesity. However, this remains to be confirmed by further studies.^[15]^

### 1.3. Genetic mechanism of obesity

Because the obesity epidemic is influenced by changes in genetic transmission involving nutritional, environmental, and lifestyle risk factors, understanding this transgenerational transmission mechanism is of special interest.^[25]^ As a result of new knowledge about epigenetics, we now know that genetic variation associated with obesity may be affected by environmental exposures that affect DNA methylation and histone modifications.^[26]^

Family and twin studies have shown that about 40–70% of obesity variation in humans is caused by genetic factors.^[27]^ Although environmental changes have increased the prevalence of obesity in the past 20 years, genetic factors play a key role in its development.^[28]^ The genome-wide association scan method has identified more than 400 genes associated with type 2 diabetes.^[29,30]^ However, these genes only predict a 5% risk of obesity.^[31]^ The low predictive power may be due to the fact that current population genetics-based approaches have not thoroughly defined the gene-gene, gene-environment, and epigenetic interactions.^[32]^ Several genes involved in obesity have been identified to be involved in the regulatory pathways of energy balance.

There are several genetic, neuroendocrine, and chromosomal precursors to obesity. Endocrine disorders such as polycystic ovary syndrome lead to an increase in body fat.^[33]^ Chromosomal defects can cause obesity, including deletions in 16pll.2, 2q37, 1p36, 9q34, 6q16, 17pll.2, and 11p13.^[34]^

We have been able to identify some of the genes responsible for the monogenic form of obesity; however, the timescale for alteration of the human genome is too long for the genome to play a major role in the current obesity pandemic. However, epigenetics may provide a relatively plausible explanation for the increasing prevalence of obesity over the past few decades without requiring radical changes in the genome.^[35]^ In multicellular organisms, the genetic code is homogeneous throughout the body; however, the expression of the code can vary among different cell types. Epigenetic studies have shown that genetically regulated changes in gene expression do not require changes in nucleotide sequence.^[36]^ Epigenetic modifications can be thought of as different packages of DNA that allow for the expression of certain genes in different tissues, and the environment and gut microbiota can influence the epigenetic programming of parental gametes or programming later in life.^[7]^ DNA methylation appears to be the most important epigenetic mechanism regulating gene expression. Altered DNA methylation may be a hallmark of many diseases such as cancer.^[37]^ Maternal metabolic status can influence DNA methylation of the clenbuterol (LEP) profile at birth, affecting metabolic remodeling in obesity.^[38]^ The epigenetic status of adiponectin has also been associated with obesity, with a reported association between LDL-cholesterol levels and DNA methylation of LEP and epigenetic status of adiponectin.^[39]^ Other genes involved in metabolism and obesity include tumor necrosis factor, hypoxia-inducible factor 3A, neuropeptide Y (NPY), insulin receptor substrate 1, mitochondrial transcription factor A, interleukin 6, lymphocyte antigen 86, and glucose transport 4.^[36]^ Histones are proteins with DNA packaging functions, and histone modifications have been implicated in the epigenetic regulation of lipogenesis and the development of obesity.^[40]^

Microribonucleic acids (miRNAs) are short non-coding RNA sequences 18 to 25 nucleotides in length that can regulate gene expression through gene silencing and post-transcriptional changes. miRNAs play a role in a variety of biological processes, including adipocyte differentiation and proliferation. It is associated with low-grade inflammation and insulin resistance in individuals.^[41]^ The levels of miRNAs, including miR-486-3p, miR-142-3p, miR-486-5p, miR-423-5p, and miR-130b, increased in children with high BMI values, and 10 showed clear changes with increasing body weight.^[42]^

### 1.4. Complexity of obesity

For clinicians treating obese patients, effective obesity management requires a systematic assessment of the factors that may affect energy intake, metabolism, and expenditure. Given the high variability in BMI among individuals sharing the same environment, it is tempting to argue that individual weight regulation has the most important effect on weight gain and should, therefore, be the target of weight loss interventions. The current weight loss strategies for individuals may not address the most important underlying causes of energy imbalance.^[43,44]^ The uncertainty of the complex causes of obesity is reflected in the Tenth Revision of the International Code of Diseases, where obesity is classified in the category of “Endocrine, Nutritional and Metabolic Diseases.”^[25]^ Although hormonal, nutritional, and metabolic factors play a role in the pathophysiology of obesity, this classification ignores other factors, including energy expenditure, psychological factors, and sedentary behavior.^[26]^ The International Code of Diseases-10 code may even be considered a stigmatization of the disease, overemphasizing the nutritional aspects of the obesity mechanism.^[26]^

### 1.5. Treatment of obesity in modern medicine

Given the lack of effective pharmacological treatments for obesity, “lifestyle modification” remains the basis of obesity treatment.^[45]^ Obese patients are advised to lose at least 60% of their body weight through a combination of diet, physical exercise, and behavioral therapy.^[46]^ Significant short-term weight loss can be achieved through diet control,^[47]^ and long-term weight control can be achieved through high levels of physical activity and sustained patient-physician contact.^[48]^

Medication is recommended for patients with BMI > 30 who are unable to lose weight through lifestyle changes alone.^[49]^ The Food and Drug Administration (FDA) has approved several new drugs for the short-term treatment of obesity. Since Lorcaserin was withdrawn, only 4 drugs have been approved, Naltrexone-BupropionUNK1 Contrave UNK2, Orlistat UNK3 Xenica, Allia UNK1, Liraglutide UNK2 Saxenda UNK3, and Phentermine-Topiramate UNK4 Qsymia UNK5. Gelesis is now the fifth approved drug for the treatment of chronic obesity.^[50,51]^

For patients with BMI > 40 or > 35 and comorbidities, bariatric surgery or bariatric surgery is an alternative if weight loss cannot be achieved through lifestyle modification or medication.^[51]^ Standard bariatric procedures, including BPD (biliopancreatic diversion), SG (sleeve gastrectomy), RYGB (Rou-en-Y gastric bypass), and AGB (adjustable gastric banding).^[52]^ Studies have reported that the benefits of bariatric surgery go beyond weight loss and can reduce chronic inflammation involving obesity, alter biomarkers, gut microbes, and cause long-term remission of type 2 diabetes.^[53,54]^

### 1.6. Recognition of obesity in Traditional Chinese Medicine

“Obesity” has been mentioned many times in the ancient classical literature of traditional Chinese medicine in the past Dynasties, which is called “fat man,” “fat noble man,” “fat body” and “cream beam.” “Su Wen · Tong Ping Xu Shi Lun” put forward that “fat people are the disease of Gao Liang,” describing it as a disease.^[55]^ “Miraculous Pivot: Defensive Qi Disorder classifies obesity according to the amount of Qi and blood in human skin and flesh, which can be divided into 3 types: “fat, cream and flesh.”^[56]^ Song · Yang Shiying “Ren Zhai Zhi Zhi Fang” points out: “Fat people have qi deficiency causing cold, cold, and dampness causing phlegm; therefore, fat people are prone to cold and dampness.^[57]^ During the Jin and Yuan Dynasties, Zhu Danxi proposed that “fat white people are more wet and “fat white people must have more phlegm.”^[58]^ Qing · Zhang Xugu also pointed out in “Yi Men Bang Drink”: “If the body is plump and white, the skin is tender and the muscle is loose, the pulse is large and scattered, although there is a lot of food, phlegm and saliva every day, this is the quality of Yin excess and Yang deficiency.” In faters, Yang qi is insufficient, yin cold is endogenous, qi does not transform into water, water dampness stops in the body, and phlegm dampness easily occurs.^[59]^

### 1.7. Etiology and pathogenesis of obesity

Traditional Chinese medicine believes that the etiology and pathogenesis of obesity are complex and varied. Its specific pathogenesis is as follows.

#### 1.7.1. Improper diet.

It is said in Treatise on the Spleen and Stomach that “when both the spleen and the stomach are strong, they can eat and become fat”.^[50,60]^ “Su Wen · Qi Bing Lun” says: “This is the origin of fat and beauty. This person must eat sweet food and be fat.” Excessive diet damages the spleen and stomach, leading to the accumulation of phlegm and dampness, abnormal lipid metabolism, and obesity.^[61]^

### 1.8. Weakness of the spleen and stomach

The spleen and stomach are subject to phlegm and dampness, forming the pathogenesis of deficiency and excess, respectively. “Yi Zong Bi Du” says: “The spleen and earth are weak, the clear is difficult to ascend, the turbid is difficult to descend, the stagnation in the diaphragm, and the phlegm is formed by blood stasis.” Spleen deficiency leads to accumulation of sputum, resulting in obesity.^[62]^

### 1.9. Deficiency of kidney-yang

Senility can lead to deficiency of kidney essence, and deficiency of kidney-yang can lead to the loss of spleen warmth. If the spleen fails to warm, transport, and transform, the grain essence will not be transported and transformed and blood stasis will accumulate in the body, resulting in obesity. Zhang Xichun pointed out in Yi Xue Zhong Zhong Shen Xi Lu: “If excessive indulgence leads to the loss of Qi and blood, the circulation of Qi and blood in the whole body is bound to be slow, and blood stasis is caused by it.” Modern studies have shown that high-density lipoprotein cholesterol content is reduced and blood lipids are easily increased due to kidney qi deficiency.^[63]^

### 1.10. Stagnation of liver-qi

Professor Zheng Shaozhou believes that qi stagnation and blood stasis are caused by the disorder of 7 emotions, the disorder of qi movement and the obstruction of the clear orifices, and the failure of the liver to disperse. Stagnation of qi and blood stasis accumulate to form phlegm and dampness, causing abnormal metabolism in the body and obesity.^[64]^

Traditional Chinese medicine believes that many factors, such as improper diet, weakness of the spleen and stomach, deficiency of kidney-yang, stagnation of liver qi, dysfunction of viscera, retention of water and dampness, accumulation of dampness in phlegm, and accumulation of phlegm in the body, result in obesity. The disease is mostly caused by a deficiency in origin and excess superficiality. The basic pathogenesis is deficiency of spleen qi and the excessive accumulation of phlegm and dampness. The disease is mainly located in the spleen and stomach, which is closely related to the kidney and involves the liver, spleen, and heart.^[65]^

### 1.11. Traditional Chinese Medicine prescription for obesity

According to the Consensus on Traditional Chinese Medicine (TCM) Diagnosis and Treatment of Obesity issued by the Chinese Society of Traditional Chinese Medicine, obesity is divided into 4 syndromes: spleen deficiency and dampness retention syndrome, gastrointestinal damp-heat syndrome, liver depression and qi stagnation syndrome, and spleen and kidney yang deficiency syndrome.^[66]^ Clinically, it is also based on these syndromes, syndrome differentiation and treatment, and medication guidance.

Yang et al designed a randomized controlled trial to explore and observe the clinical effects of Jiawei Cangfu Daotan Decoction (Rhizoma Atractylodis, Rhizoma Cyperi, Pericarpium Citri Reticulatae, Radix Salviae Miltiorrhizae, Rhizoma Pinelliae, Fructus Aurantii, Gynostemma Pentaphyllum, Poria, Massa Medicata Fermentata, Folium Nelumbinis, Arisaema Cum Bile, and Fructus Crataegi). 72 cases of phlegm-dampness-type simple obesity with dyslipidemia were selected. The patients were divided into treatment and control groups according to a random number table, with 36 cases in each group. The control group received basic treatment plus metformin hydrochloride tablets, and the treatment group was given modified Cangfu Daotan Decoction on the basis of the control group. Both the groups were treated for 12 weeks. BMI, abdominal circumference, insulin resistance index (HOMA-IR), blood lipid level, TCM syndrome score, and clinical efficacy were assessed before and after treatment. The study found that Jiawei Cangfu Daotan Decoction can reduce weight and waist circumference, regulate blood lipid metabolism, alleviate insulin resistance, and improve clinical symptoms in patients with phlegm-dampness-type simple obesity with dyslipidemia.

Wang et al designed a randomized controlled trial to explore the efficacy of Jianpi Xiaotan Fang (Jianghoupu, Pinellia, Atractylodes, Atractylodes macrocephala, Chigencao, Tangerine Peel, Poria, Agastache rugosa, Fructus Aurantii Immaturus, Radix Glycyrrhizae Preparata, and Saffron) in treating obese patients with spleen deficiency and dampness retention, and its effect on glucose and lipid metabolism.^[68]^ Sixty obese patients with spleen deficiency and dampness retention were randomly divided into 2 groups, with 30 cases in each group. The control group received routine healthy diet and exercise education guidance, while the observation group was given Jianpi Xiaotan Decoction based on intervention in the control group. TCM symptom scores (abdominal fullness, obesity, heavy limbs, anorexia, and fatigue), BMI, waist-hip ratio (WHR), blood glucose indicators (fasting blood glucose, 2 H postprandial blood glucose), and fat metabolism indicators (triglyceride (TG), total cholesterol (TC), and low-density lipoprotein cholesterol) were observed and compared between the 2 groups after 3 months of treatment. High-density lipoprotein cholesterol level, clinical total effective rate, and adverse reactions. The study found that Jianpi Xiaotan Prescription can regulate the metabolism of glucose and lipids in patients with obesity of spleen deficiency and dampness retention type and effectively reduce the body weight of the patients, which is safe.

Liu et al designed a randomized controlled trial to explore the clinical efficacy of Qiwei Baizhu Powder (Radix Pseudostellariae, Rhizoma Atractylodis Macrocephalae, Poria, Herba Agastaches, Radix Aucklandiae, Radix Puerariae, and Radix Glycyrrhizae Preparata) in the treatment of obesity with dampness retention due to spleen deficiency.^[69]^ 34 cases Thirty-four obesity patients with dampness retention due to spleen deficiency were randomly divided into treatment (24 cases) and control (10 cases) groups. Patients in the control group were provided lifestyle guidance, regular diet control, and proper exercise intervention. The patients in the treatment group were orally administered Qiwei Baizhu Powder based on the treatment in the control group, 1 dose per day. The course of treatment was 3 months in both groups. The TCM syndrome score, BMI, blood lipid (TG, LDL, HDL), glycometabolism indicators (fasting blood glucose, fasting insulin, glycosylated hemoglobin, and insulin resistance index (HOMA-IR)), and intestinal flora distribution were compared between the 2 groups before and after treatment. The study found that Qiwei Baizhu Powder can effectively regulate the distribution of intestinal flora and improve the metabolism of blood sugar and lipids in obese patients with spleen deficiency and dampness retention.

Liu et al designed a meta-analysis to systematically evaluate the effectiveness and safety of traditional Chinese compounds in the treatment of obesity.^[70]^ CNKI, VIP, WanFang Data, CBM, PubMed, Cochrane, AMED, CINAHL, ClinicalTrials, Embase, Informit, ProQuest, SciFinder, Scopus, Web of Science, and other Chinese and English databases contain relevant literature (randomized controlled trials) on the treatment of obesity with traditional Chinese compounds, which were published from the establishment of the database to July 30, 2022. The Cochrane Risk of Systematic Bias Tool was used to assess the quality of the literature, and the meta-analysis was performed using the RevMan 5.4 software. The study found that Chinese herbal compounds are an effective and safe method for the treatment of obesity, and the ideal application mode is based on classical prescriptions and syndrome differentiation and treatment.

### 1.12. Treatment of obesity with acupuncture

Si Yuancheng et al designed animal experiments to explore the effects of electroacupuncture (Tianshu, Guanyuan, Zusanli and Sanyinjiao) on adipokines and fatty acid synthase in nutritionally obese rats.^[71]^ In the experiment, 50 SPF C57BL/6 mice were used, 6 of which were selected as the normal group by the random number table method, and the remaining 44 were used to establish a nutritional obesity mouse model. After 8 weeks, 18 obese rats whose body mass was 20% larger than that of the normal group were selected and randomly divided into the model, 14-day electroacupuncture, and 28-day electroacupuncture groups, with 6 rats in each group. Changes in body weight, Lee index, serum lipids (CHO and TG), adipokines (LP and ADPN), and fatty acid synthase in fat and liver tissues were observed. The study found that electroacupuncture can correct dyslipidemia in mice with nutritional obesity, regulate the metabolic function of fatty acid synthase, and achieve weight loss.

Luo et al designed a randomized controlled trial to observe the effect of acupoint catgut embedding (Feishu, Pishu, Sanjiaoshu, Mingmen, Bladder Shu, Shanzhong, Zhongwan, Qihai, Tianshushuang, Zusanli and Xuehai) on NPY and adiponectin (ADP) in patients with simple obesity.^[72]^ The 58 cases of obesity were randomly divided into a catgut embedding therapy group (28 cases) and a Western medicine group (30 cases). The 2 groups were treated with catgut embedding therapy to regulate the triple energizer and metformin orally. After treatment, changes in obesity indicators, such as BMI, waist circumference, WHR, NPY, and ADP were observed, and their clinical significance was analyzed. The study found that the method of regulating triple energizer and acupoint catgut embedding is an effective method for the treatment of obesity, which is superior to the Western medicine metformin, and can benignly regulate the levels of serum NPY and ADP, thus possibly improving the mechanism of leptin resistance and insulin resistance to achieve the effect of weight loss.

Qian Yani et al designed a randomized controlled trial to explore the effect of electroacupuncture combined with acupoint catgut embedding (Zhongwan, Tianshu, Qihai, Shuidao, Daimai and Zusanli) on obesity indicators and body composition of patients with simple obesity.^[73]^ Ninety patients with simple obesity were randomly treated with electroacupuncture (electroacupuncture group), acupoint catgut embedding (catgut embedding group), or electroacupuncture plus acupoint catgut embedding (electroacupuncture catgut embedding group), and the obesity index, body composition index, and curative effect of each group were compared after treatment. The study found that electroacupuncture combined with acupoint catgut embedding therapy has a synergistic effect on simple obesity and is superior to simple electroacupuncture or acupoint catgut embedding therapy.

Liang et al designed clinical observations to observe the clinical efficacy of warming and dredging acupuncture (Zhongwan, Tianshu, Daimai, Shuifen, Qihai, Pishu, Zhangmen, Zusanli, Yinlingquan, Fenglong, Taibai) in the treatment of simple obesity of spleen deficiency and dampness retention.^[74]^ One hundred and twenty patients with simple obesity of spleen deficiency and dampness retention type were randomly divided into twirling, lifting and thrusting, and warming and unblocking acupuncture groups, with 40 cases in each group. The twirling group was treated with acupuncture manipulation using twirling reinforcement. The lifting and thrusting groups were treated with lifting and thrusting acupuncture manipulations. The warming and unblocking acupuncture groups were treated with warming and unblocking acupuncture, respectively. Clinical efficacy, blood lipid levels (TC, TG, and HDL-C), and clinical safety of the 3 groups were observed. The study found that warming and unblocking needling therapy is effective, safe, and reliable in treating simple obesity due to spleen deficiency and dampness retention.

Wang et al designed a meta-analysis to systematically evaluate the clinical efficacy of electroacupuncture in the treatment of simple obesity over the past 20 years.^[75]^ The systematic review comprehensively searched the published literature on randomized controlled trials of electroacupuncture in the treatment of simple obesity in China, screened the literature that met the inclusion criteria, and systematically analyzed body weight, BMI, waist circumference, hip circumference, and other indicators using Rev Man 5.3. Fifteen studies involving 903 patients were included. The study found that the clinical efficacy of electroacupuncture in the treatment of simple obesity is good, but due to the low quality of the literature, large and high-quality research samples are still needed for verification.

### 1.13. Treatment of obesity with combination of acupuncture and medicine

Han et al designed clinical observations to observe Jianpi Tiaogan Yin (Radix Astragali, Poria, Radix Paeoniae Alba, Radix Bupleuri, Radix Salviae Miltiorrhizae, Rhizoma Alismatis, Rhizoma Atractylodis Macrocephalae, Herba Eupatorii, Semen Cassiae, Radix et Rhizoma Rhei Preparata, and Fructus Crataegi) combined with Yiyi navel acupuncture [with navel pistil as the center, 3 days a week, select Zhen (9 o’clock), Xun (10–11 o’clock), Select Kun (1–2 points), Dui (3 points), and Kan (6 points) to insert the needle in sequence on the 2nd day, rest on the 2nd day, and insert the needle with reference to the 8 diagrams of the umbilicus^[76]^ for the clinical efficacy of simple obesity of liver depression and spleen deficiency type.^[77]^ Sixty-seven patients with simple obesity, liver depression, and spleen deficiency type were randomly divided into 3 groups: traditional Chinese medicine group (n = 23), umbilical acupuncture group (n = 22), and acupuncture and medicine group (n = 22). The patients in the 3 groups were given healthy life guidance, and the traditional Chinese medicine group was treated with Jianpi Tiaogan Decoction. In the navel acupuncture group, treatment was centered on the umbilicus. The acupuncture group was treated with the Jianpi Tiaogan Decoction combined with navel acupuncture. The treatment lasted for 7 days, with a total of 8 courses. After 8 weeks of treatment, changes in body mass, BMI, waist circumference, and WHR before and after treatment were observed, as well as changes in 4 blood lipid levels. Changes in TCM syndrome scores before and after treatment were compared among the 3 groups. The occurrence of adverse reactions in the 3 groups was evaluated. The study found that Jianpi Tiaogan Decoction combined with Yiyi navel acupuncture treatment of liver depression and spleen deficiency-type simple obesity can significantly reduce the body mass and other related indicators, improve the level of blood lipid metabolism, and improve the clinical symptoms of liver depression and spleen deficiency, thereby improving the quality of life of patients.

Liu Juanjuan et al designed a randomized controlled trial to observe the effect of Shabanlizhong Decoction (Radix Ginseng Rubra, Rhizoma Atractylodis Macrocephalae, Rhizoma Zingiberis, Rhizoma Pinelliae, Fructus Amomi, and Radix Glycyrrhizae Preparata) combined with acupuncture at 6 fu organs and mu points (single side of Zhongwan, Guanyuan, and Zhongji; Bilateral Zusanli, Xiajuxu, Shangjuxu, Weizhong, Weiyang, Yanglingquan, Tianshu, Riyue and Shimen) on the body mass index and physical and mental health of patients with simple obesity.^[78]^ A total of 99 patients with simple obesity were enrolled in the study and randomly divided into 2 groups: 44 patients in the control group were given conventional clinical intervention, and 45 patients in the observation group were given Shabanlizhong Decoction combined with acupuncture at 6 fu-organs and mu points on the basis of conventional intervention. Clinical efficacy, integral changes in TCM symptoms (obesity, edema, night sweat, shortness of breath, heavy limbs, fatigue, and weakness), changes in body mass, body mass index (BMI), waist circumference, subcutaneous fat thickness (midpoint of the lateral arm of the lower edge of the right deltoid muscle, lower corner of the right scapula, side of the right umbilicus, and anterior superior spine of the right ilium), and blood lipid levels were compared between the 2 groups before and after the intervention. Changes in the Self-rating Anxiety Scale, Self-rating Depression Scale, quality of life scale, and Satisfaction. The study found that Shaban Lizhong Decoction combined with acupuncture at 6 fu organs and multiple points can improve the therapeutic effect of simple obesity patients, improve the symptoms of patients, have a positive impact on the physical and mental health of patients, improve the psychological state of patients, improve the quality of life, and have a high satisfaction rate, which is worthy of promotion and application.

Zhu Ranran et al designed a randomized controlled trial to explore Professor Chen Qiu self-made comprehensive prescription (Astragalus membranaceus, Poria cocos, Atractylodes macrocephala, Alisma orientale, Pericarpium citri reticulatae, Ligusticum chuanxiong, Salvia miltiorrhiza, raw hawthorn, wine rhubarb) combined with acupoint catgut embedding (the main acupoints of catgut embedding were divided into 2 groups, the first group was Tianshu, Zhongwan, Guanyuan, Zusanli, Fenglong For each treatment, acupoints were selected in single group and double groups alternately.) To observe the clinical efficacy in the treatment of obesity with spleen deficiency and dampness retention type,^[79]^ seventy-four obese patients with spleen deficiency and dampness retention were randomly divided into a control group (n = 38) and an observation group (n = 36). In addition to the basic treatment, the control group was treated with Dai Zong Fang and metformin, while the observation group was treated with Dai Zong Fang and acupoint catgut embedding. The drug treatment cycle was 12 weeks. Physical examination indexes, relevant laboratory detection indexes, TCM syndrome scores, and clinical efficacy were compared between the 2 groups before and after treatment. The study found that the treatment of Dai Zong Fang combined with acupoint catgut embedding can significantly improve clinical symptoms, reduce body weight, increase insulin sensitivity, and improve insulin resistance in obese patients with spleen deficiency and dampness retention.

## 2. Summary

To date, there is no effective drug treatment for obesity in modern medicine,^[45]^ the efficacy of the approved drugs is not satisfactory, and the side effects are relatively high, which is harmful to the human body. However, the surgical treatment of obesity has the disadvantages of vague surgical indications and abuse of surgery. Its adverse effects and harmful effects on the human body are also obvious. The combination of acupuncture and traditional Chinese medicine has remarkable curative effects and is safe for the treatment of obesity. A systematic review pointed out that on the basis of a reasonable diet and proper exercise, acupuncture is safe and effective in the treatment of simple obesity, which may be superior to conventional Western medicine. Due to the limited number of included studies and the low quality of some of them, more high-quality randomized controlled studies are needed for further verification.^[80]^ According to a recent meta-analysis, Chinese medicine treatment of obesity has a certain effect in reducing weight and improving lipid metabolism and related syndromes.^[55]^ Another systematic review pointed out that, equivalent to the modern medical drug treatment model, Chinese medicine compounds are an effective method for the treatment of obesity, with good safety, and the ideal application model is prescriptions based on classical prescriptions and syndrome differentiation.^[70]^

Obesity has a complex etiology, complex conditions, and diverse symptoms, involving multiple viscera and organs, and is accompanied by a variety of metabolic disorders. The treatment mode of acupuncture combined with traditional Chinese medicine can give full play to the advantages of multi-center and multi-target treatment, so that its overall efficacy is better than that of a single-drug treatment mode. However, compared with the seriousness of the global obesity problem, research on obesity in traditional Chinese medicine is far from sufficient, especially regarding its molecular mechanism. The Chinese medicine community should: With the help of a variety of cash detection technologies, combined with genomics, proteomics, metabolomics, and other multi-group molecular mechanisms, the scientific theory of TCM syndrome differentiation, compatibility of traditional Chinese medicine, and the combination of acupuncture and medicine is analyzed. To carry out multi-center and large-sample cohort studies to improve the quality of research on the treatment of obesity with acupuncture and medicine. It is expected that academic circles will produce more gratifying research results on obesity in the future and continuously improve the level of human understanding of obesity and its treatment methods.

This study was a preliminary exploration of obesity by the authors’ team. In the next step, the author team will collect published literature on the treatment of obesity in traditional Chinese medicine and conduct a systematic review.

## Author contributions

**Conceptualization:** Shiyu Niu, Lihong Ren.

**Data curation:** Shiyu Niu, Lihong Ren.

**Formal analysis:** Shiyu Niu.

**Funding acquisition:** Shiyu Niu.

**Investigation:** Shiyu Niu.

**Methodology:** Shiyu Niu.

**Project administration:** Shiyu Niu.

**Resources:** Shiyu Niu.

**Supervision:** Shiyu Niu.

**Validation:** Shiyu Niu.

**Writing – original draft:** Shiyu Niu.

**Writing – review & editing:** Shiyu Niu.
